# Can clinically relevant prognostic subsets of breast cancer patients with four or more involved axillary lymph nodes be identified through immunohistochemical biomarkers? A tissue microarray feasibility study

**DOI:** 10.1186/bcr1847

**Published:** 2008-01-14

**Authors:** Simon J Crabb, Chris D Bajdik, Samuel Leung, Caroline H Speers, Hagen Kennecke, David G Huntsman, Karen A Gelmon

**Affiliations:** 1 Department of Medical Oncology, BC Cancer Agency, 600 West 10th Avenue, Vancouver, BC, Canada, V5Z 4E6; 2 Cancer Control Research Program, BC Cancer Agency, 675 West 10th Avenue, Vancouver, BC, Canada, V5Z 1L3; 3 Genetic Pathology Evaluation Centre, University of British Columbia, 600 West 10th Avenue, Vancouver, BC, Canada, V5Z 4E6; 4 Breast Cancer Outcomes Unit, BC Cancer Agency, 600 West 10th Avenue, Vancouver, British Columbia, Canada, V5Z 4E6

## Abstract

**Introduction:**

Primary breast cancer involving four or more axillary lymph nodes carries a poor prognosis. We hypothesized that use of an immunohistochemical biomarker scoring system could allow for identification of variable risk subgroups.

**Methods:**

Patients with four or more positive axillary nodes were identified from a clinically annotated tissue microarray of formalin-fixed paraffin-embedded primary breast cancers and randomized into a 'test set' and a 'validation set'. A prospectively defined prognostic scoring model was developed in the test set and was further assessed in the validation set combining expression for eight biomarkers by immunohistochemistry, including estrogen receptor, human epidermal growth factor receptors 1 and 2, carbonic anhydrase IX, cytokeratin 5/6, progesterone receptor, p53 and Ki-67. Survival outcomes were analyzed by the Kaplan–Meier method, log rank tests and Cox proportional-hazards models.

**Results:**

A total of 313 eligible patients were identified in the test set for whom 10-year relapse-free survival was 38.3% (SEM 2.9%), with complete immunohistochemical data available for 227. Tumor size, percentage of positive axillary nodes and expression status for the progesterone receptor, Ki-67 and carbonic anhydrase IX demonstrated independent prognostic significance with respect to relapse-free survival. Our combined biomarker scoring system defined three subgroups in the test set with mean 10-year relapse-free survivals of 75.4% (SEM 7.0%), 35.3% (SEM 4.1%) and 19.3% (SEM 7.0%). In the validation set, differences in relapse-free survival for these subgroups remained statistically significant but less marked.

**Conclusion:**

Biomarkers assessed here carry independent prognostic value for breast cancer with four or more positive axillary nodes and identified clinically relevant prognostic subgroups. This approach requires refinement and validation of methodology.

## Introduction

Prognostic assessment for early breast cancer in the clinic is currently made from clinical and pathological parameters, which at present include three biomarkers: estrogen receptor (ER), progesterone receptor (PR) and human epidermal growth factor receptor 2 (HER2) [1-3]. Of these conventional prognostic factors, nodal status is consistently held to be the most important parameter for determining prognosis [3-5]. The widely referenced St Gallen consensus guidelines, for primary therapy of early breast cancer, define patients with four or more positive axillary nodes as 'high risk' irrespective of the status of any other prognostic factor [5]. From the perspective of recommendations for the use of adjuvant chemotherapy, the presence of four or more positive axillary lymph nodes defines all such patients into a group offered treatment regardless of other conventional parameters aside from performance status and age [5,6].

It has become clear that breast cancer is in fact a collection of heterogeneous disease processes, with variable biological behavior and outcome, that current models for prognostication do not completely capture [2,7-12]. Protein or mRNA expression profiling has been shown to permit the molecular classification of breast cancers via a range of techniques including cDNA microarray, quantitative RT-PCR and tissue microarray (TMA) into consistently observable groupings [7-10,12-19]. Each of these approaches provides prognostic information through a molecular subtype classification of breast cancer, but there is less evidence as to how these approaches compare or add to the use of conventional prognostic factors [7-10,14,17,18]. The potential to use such methodologies, in the setting of axillary lymph node negative breast cancer, to inform the decisions regarding chemotherapy is currently being tested in prospective randomized trials [1,17-21].

We hypothesized that TMA profiling of a panel of biomarkers, either proven or potentially relevant for prognostic and/or predictive assessment of breast cancer, might permit the detection of clinically relevant prognostic groups from those with four or more positive axillary lymph nodes above that attainable from conventional factors alone. Such information might be helpful in providing treatment recommendations and prognosis but might also be helpful in the design and stratification of patients on clinical trials.

## Materials and methods

### Study population

The study population was derived from a TMA constructed from archival formalin-fixed paraffin-embedded specimens of 4,444 patients from the Canadian province of British Columbia. All patients had been diagnosed with invasive breast cancer without metastatic disease between 1986 and 1992, and represented 34% of patients diagnosed with breast cancer during this period [22]. Clinical and pathological information was collected prospectively through the Breast Cancer Outcomes Unit Database of the British Columbia Cancer Agency. Patients were randomly allocated into two groups of 2,222 after stratification for treatment. Inclusion criteria for this study were: female sex, known cause of death, new breast cancer diagnosis at the time of referral to the British Columbia Cancer Agency, and a known number of positive axillary lymph nodes. From this set, those patients with four or more positive axillary nodes formed the final cohorts. The 'test set' was used to define prognostic subgroups based on patterns of immunohistochemical biomarker expression. The prognostic value of the biomarker-derived subgroups was then further evaluated in the 'validation set'. The study was approved by the Clinical Research Ethics Board of the University of British Columbia.

### Tissue microarray, immunohistochemistry and biomarker scoring

TMAs were constructed as described previously, requiring 17 TMA blocks [22]. TMA slides were stained for eight biomarkers by immunohistochemistry. ER (SP1, dilution 1:250), HER2 (SP3, dilution 1:100) and Ki-67 (SP6, dilution 1:200) were from Lab Vision (Fremont, CA, USA). Human epidermal growth factor receptor 1 (EGFR; PharmDx Kit, undiluted) and p53 (DO-7, dilution 1:400) were from Dako Corporation (Carpinteria, CA, USA). PR (1E2, undiluted) was from Ventana Medical Systems (Tucson, AZ, USA). Cytokeratin 5/6 (CK5/6; D5/16B4, dilution 1:100) was from Zymed Laboratories (San Francisco, CA, USA). Carbonic anhydrase IX (CA IX; M75, dilution 1:50) was a gift from Dr Stephen Chia (British Columbia Cancer Agency, BC, Canada) [23]. Biomarkers were chosen for known prognostic, and in some cases predictive, effect and relevance to biologic classification of subtypes. There were no assumptions about which would be of value for the detection of patients with good versus poor prognosis in the study cohort. Cut points to dichotomize outcome were defined prospectively as follows. ER, <1% versus ≥1% nuclei stained; PR, <1% versus ≥1% nuclei stained; EGFR, negative versus any staining; Ki-67, <10% versus ≥10% positive nuclei; p53, ≤10% versus >10% positive nuclei; CA IX, negative versus tumor and/or stroma positive; CK5/6, negative versus any staining. For HER2, TMA slides were scored by using the immunohistochemical HercepTest (Dako Corporation) scoring system. Cases with a HER2 HercepTest score of 3 were scored as positive, and those of 0 or 1 were scored as negative. Those cases with HER2 HercepTest score of 2 were re-evaluated by using fluorescence *in situ *hybridization (FISH) assays, and only those cases with a HER2 FISH amplification ratio of at least 2.0 were scored as HER2 positive.

The full set of eight biomarkers were not available for all patients as a result of tissue cores falling off slides during processing, insufficient or absent tumor tissue within cores, or artefactual distortion of the tissue making interpretation impossible. Stained TMA slides were digitally scanned and linked to a relational database [22,24]. For each biomarker, images were scored visually by two pathologists, blinded to clinical outcome. An internet website was then constructed from this database by using a WebSlide-Viewer Java applet provided by the manufacturer to view the microarray images and to permit an image-zooming functionality. This website is publicly accessible [25].

### Statistical analysis and result validation

Statistical analysis was performed with SPSS software, version 13.0 (SPSS Inc, Chicago, IL, USA). Univariate analysis of relapse-free and overall survival was performed with the Kaplan–Meier method, with survival differences analyzed by log rank tests. Cox proportional-hazards models were used to determine hazard ratios in univariate and multivariate analyses. *P *< 0.05 was considered statistically significant. The primary outcome measure for this study was of relapse-free survival (RFS); the secondary outcome measure was overall survival (OS). RFS was defined as the time from the date of diagnosis to either the first local, regional or distant recurrence or death from breast cancer before a recorded relapse. OS was calculated from the time of diagnosis to death from any cause. We used a split-sample validation technique for statistical analysis, as described previously [22]. In brief, a large data collection (*n *= 4,444) was randomly split into a 'test' set and a 'validation' set, each containing 2,222 observations. After exploratory analyses with the test set, selected final analyses were repeated with the validation set. Analyses with the validation set were undertaken by a different investigator from those using the test set.

### Determination of mean predicted relapse-free survival outcomes

Ten-year outcomes for RFS were determined by Kaplan–Meier analysis for the test set for the overall eligible cohort and prognostic subgroups defined in this study. These were compared with the means of the predicted RFS values for each patient with respect to these same subgroups provided by the online breast cancer prognostic tool Adjuvant! (version 8.0, accessed 29 December 2006) [26-28]. In determining predicted outcomes by Adjuvant! for each patient, a default option of 'average for age' was selected for the 'comorbidity' data entry point. Data for age, pathological ER status, tumor grade, tumor size, number of positive axillary nodes (four to nine versus ten or more), and type of hormonal therapy and chemotherapy used were inputted from abstracted clinical and pathological details.

## Results

In the test set, the number of positive axillary nodes was known for 2,115 patients. Of these, 325 had four or more positive axillary lymph nodes, from which 313 met the remaining eligibility criteria for inclusion. Scoring was possible for all eight of the biomarkers assessed for 227 of these 313 patients. Baseline clinical, pathological and treatment details are shown in Table 1 for the 313-patient overall test set cohort and for the 227-patient subgroup with complete biomarker scores. The 227-patient subgroup did not differ from the 313-patient overall group with respect to median RFS (5.2 years (95% confidence interval (CI) 3.6 to 6.9) and 5.2 years (3.9 to 6.5), respectively) or overall survival (6.6 years (5.2 to 8.0) and 6.7 years (5.7 to 7.8), respectively).

**Table 1:** Frequencies of conventional prognostic factors and adjuvant treatments in the test set

Factor	Division	Whole set (313 patients)		Subset (227 patients)	
		*n*	Percentage	*n*	Percentage
Age	≤40	34	10.9	25	11.0
	>40	279	89.1	202	89.0
Menopausal status	Pre	104	33.2	76	33.5
	Post	203	64.9	147	64.8
	Unknown	6	1.9	4	1.8
Tumor grade	1	5	1.6	4	1.8
	2	101	32.3	71	31.3
	3	191	61.0	144	63.4
	Unknown	16	5.1	8	3.5
Tumor size, cm	0–2	100	31.9	71	31.3
	> 2–5	173	55.3	131	57.7
	> 5	35	11.2	22	9.7
	Unknown	5	1.6	3	1.3
Pathological ER status	Negative	72	23.0	54	23.8
	Positive	234	74.8	169	74.4
	Unknown	7	2.2	4	1.8
Lymphovascular invasion	Negative	49	15.7	33	14.5
	Positive	244	78.0	184	81.1
	Unknown	20	6.4	10	4.4
Percentage of positive nodes	0–50	121	38.7	89	39.2
	>50–99.9	142	45.4	95	41.9
	100	50	16.0	43	18.9
Number of positive nodes	4–9	235	75.1	175	77.1
	≥10	78	24.9	52	22.9
Histology	Ductal	279	89.1	210	92.5
	Lobular	34	10.9	17	7.5
Adjuvant chemotherapy	No	155	49.5	107	47.1
	Yes	158	50.5	120	52.9
Adjuvant hormonal therapy	No	104	33.2	78	34.4
	Yes	209	66.8	149	65.6
Adjuvant radiotherapy	No	70	22.4	50	22.0
	Yes	243	77.6	177	78.0
Mastectomy	No	2	0.6	1	0.4
	Complete	217	69.3	154	67.8
	Partial	94	30.0	72	31.7

Data are presented for the 313-patient test set cohort, and for the 227-patient subgroup in which expression data were available for each of the eight immunohistochemical biomarkers assessed. Percentages are rounded.

Univariate analysis of conventional prognostic markers was performed with respect to RFS in the test set (Table 2). Increasing tumor grade (grade 3 versus 1 or 2), increasing tumor size, negative baseline pathological ER status, presence of lymphovascular invasion and increasing percentage of positive axillary nodes were predictive of inferior outcome with respect to RFS. In multivariate Cox regression analysis, baseline pathological ER status (*P *= 0.0005) and tumor size (*P *= 0.03) retained prognostic significance (Table 3).

**Table 2:** Univariate analysis of relapse-free survival for conventional prognostic factors in the test set cohort

Factor	*n*/313	Divisions	*n*	Median RFS, years (95% CI)	HR (95% CI)	*P*
Overall	313			5.2 (3.9–6.5)		
Age	313	≤40	34	2.9 (1.3–4.5)	1	
		>40	279	5.6 (4.2–7.0)	0.74 (0.48–1.13)	0.2
Menopausal status	307	Pre	104	3.7 (2.1–5.2)	1	
		Post	203	6.0 (3.9–8.2)	0.79 (0.59–1.05)	0.1
Tumor grade	297	1, 2	5, 101	9.5 (4.9–14.2)	1	
		3	191	4.0 (3.3–4.8)	1.24 (1.06–1.45)	0.007
Tumor size, cm	308	0 to 2	100	11.0	1	
		>2 to 5	173	4.5 (3.5–5.6)	1.67 (1.21–2.33)	0.002
		>5	35	3.0 (0–6.2)	1.55 (0.95–2.54)	0.08
Pathological ER status^a^	306	Negative	72	2.0 (0.9–3.0)	1	
		Positive	234	6.8 (4.0–9.5)	0.53 (0.38–0.72)	0.00007
Lymphovascular invasion	293	Negative	49	12.0 (5.3–18.8)	1	
		Positive	244	4.5 (3.5–5.4)	1.73 (1.13–2.66)	0.01
Percentage of positive nodes	313	0–50	121	9.2 (5.7–12.8)	1	
		>50–99	142	4.8 (3.5–6.2)	1.52 (1.11–2.09)	0.01
		100%	50	3.1 (1.8–4.4)	1.96 (1.30–2.94)	0.001
Number of positive nodes	313	4–9	235	5.3 (3.9–6.7)	1	
		≥10	78	4.8 (2.1–7.6)	1.16 (0.85–1.60)	0.4
Histology	313	Ductal	279	5.1 (3.8–6.5)	1	
		Lobular	34	6.4 (2.7–10.1)	1.16 (0.76–1.77)	0.5

*P*, significance for the comparison of hazard ratios; ER, estrogen receptor; RFS, relapse-free survival; CI, confidence interval; HR, hazard ratio.

^a^Pathological ER status at diagnosis.

**Table 3:** Multivariate analysis for relapse-free survival in the test set cohort of baseline prognostic factors

Factor	Hazard ratio (95% CI)	*P*
Grade (3 versus 1 or 2)	1.20 (0.84–1.70)	0.3
Tumor size, cm		0.03
>2–5 versus 0–2	1.62 (1.12–2.33)	0.01
>5 versus 0–2	1.23 (0.71–2.12)	0.5
Pathological ER status^a^	0.53 (0.37–0.76)	0.0005
Lymphovascular invasion	1.32 (0.84–2.09)	0.2
Percentage of positive nodes		0.09
>50–99 versus 0–50	1.22 (0.87–1.73)	0.3
100 versus 0–50	1.62 (1.05–2.50)	0.03

Multivariate Cox regression analysis was performed inclusive of those factors reaching statistical significance in univariate analysis. ER, estrogen receptor; CI, confidence interval.

^a^Pathological ER status at diagnosis.

The prognostic value of eight biomarkers determined by immunohistochemistry with TMA was assessed. In univariate analysis within the test set (Table 4), increased expression of EGFR, Ki-67, p53 and CA IX, and lower expression of ER and PR, indicated poorer prognosis with respect to RFS. Increased expression of HER2 and CK5/6 did not significantly predict outcomes. In multivariate Cox regression analysis inclusive of all eight biomarkers, PR (*P *= 0.006), Ki-67 (*P *= 0.001) and CA IX (*P *= 0.03) retained independent prognostic significance in the test set (Table 5).

**Table 4:** Univariate analysis of relapse-free survival for immunohistochemical biomarkers in the test and validation sets

Set	Biomarker	+/-	*n*/total	*n*	Median RFS, years (95% CI)	HR (95% CI)	*P*
Test	ER	-	308/313	100	2.9 (1.7–4.1)	1	
		+		208	6.1 (4.1–8.1)	0.70 (0.52–0.93)	0.02
	PR	-	279/313	143	3.0 (2.1–3.8)	1	
		+		136	8.2 (5.0–11.4)	0.58 (0.43–0.78)	0.0003
	HER2	-	300/313	240	6.0 (4.4–7.6)	1	
		+		60	3.6 (2.1–5.1)	1.33 (0.94–1.89)	0.1
	EGFR	-	271/313	239	6.0 (4.0–8.0)	1	
		+		32	1.6 (0.9–2.3)	2.30 (1.49–3.56)	0.0002
	Ki-67	-	308/313	140	11.0 (6.5–15.6)	1	
		+		168	3.6 (2.8–4.4)	1.93 (1.44–2.60)	0.00001
	p53	-	307/313	235	6.4 (4.3–8.6)	1	
		+		72	2.6 (1.9–3.3)	1.59 (1.16–2.18)	0.004
	CA IX	-	281/313	234	5.7 (4.0–7.4)	1	
		+		47	2.5 (1.4–3.6)	1.81 (1.26–2.62)	0.002
	CK5/6	-	260/313	238	5.2 (3.8–6.6)	1	
		+		22	2.5 (0.6–4.3)	1.60 (0.94–2.73)	0.08
Validation	ER	-	288/289	100	2.6 (1.5–3.8)	1	
		+		188	7.2 (5.8–8.5)	0.68 (0.50–0.92)	0.01
	PR	-	268/289	143	3.8 (2.1–5.5)	1	
		+		125	7.2 (6.1–8.3)	0.73 (0.54–1.00)	0.05
	HER2	-	273/289	206	7.0 (5.8–8.2)	1	
		+		67	2.4 (1.5–3.2)	1.55 (1.11–2.18)	0.01
	EGFR	-	257/289	219	6.6 (4.9–8.3)	1	
		+		38	2.1 (1.3–2.8)	1.81 (1.21–2.70)	0.004
	Ki-67	-	289/289	134	7.2 (5.6–8.8)	1	
		+		155	4.8 (3.0–6.6)	1.27 (0.94–1.70)	0.12
	p53	-	286/289	223	6.7 (5.1–8.2)	1	
		+		63	2.7 (0.5–4.9)	1.40 (0.99–1.97)	0.06
	CA IX	-	271/289	227	7.1 (5.4–8.6)	1	
		+		44	2.3 (1.3–3.2)	1.79 (1.23–2.61)	0.002
	CK5/6	-	252/289	237	6.0 (4.3–7.6)	1	
		+		15	2.4 (1.7–3.1)	1.81 (1.00–3.27)	0.05

ER, estrogen receptor; PR, progesterone receptor; HER2, human epidermal growth factor receptor 2; EGFR, human epidermal growth factor receptor 1; CA IX, carbonic anhydrase IX; CK5/6, cytokeratin 5/6; RFS, relapse-free survival; CI, confidence interval; HR, hazard ratio; *P*, significance for comparison of hazard ratio.

**Table 5:** Multivariate analysis of relapse-free survival in the test set for all eight tissue microarray biomarkers

Biomarker	HR (95% CI)	*P*
ER	0.87 (0.55–1.38)	0.5
PR	0.58 (0.39–0.86)	0.006
HER2	0.93 (0.61–1.42)	0.7
EGFR	1.20 (0.63–2.29)	0.6
Ki-67	1.99 (1.32–3.00)	0.001
p53	0.88 (0.58–1.35)	0.6
CA IX	1.67 (1.06–2.64)	0.03
CK5/6	0.81 (0.43–1.54)	0.5

ER, estrogen receptor; PR, progesterone receptor; HER2, human epidermal growth factor receptor 2; EGFR, human epidermal growth factor receptor 1; CA IX, carbonic anhydrase IX; CK5/6, cytokeratin 5/6; HR, hazard ratio for positive expression status for that marker; CI, confidence interval.

Univariate analysis of RFS outcomes was repeated for the same eight biomarkers within the validation set (Table 4). In this cohort, 289 had four or more positive axillary lymph nodes and met the eligibility criteria, with 219 having data for all eight biomarkers for analysis. Biomarkers reaching statistical significance with respect to RFS in the validation set were ER, PR, HER2 EGFR, CA IX and CK5/6.

**Figure 1:**
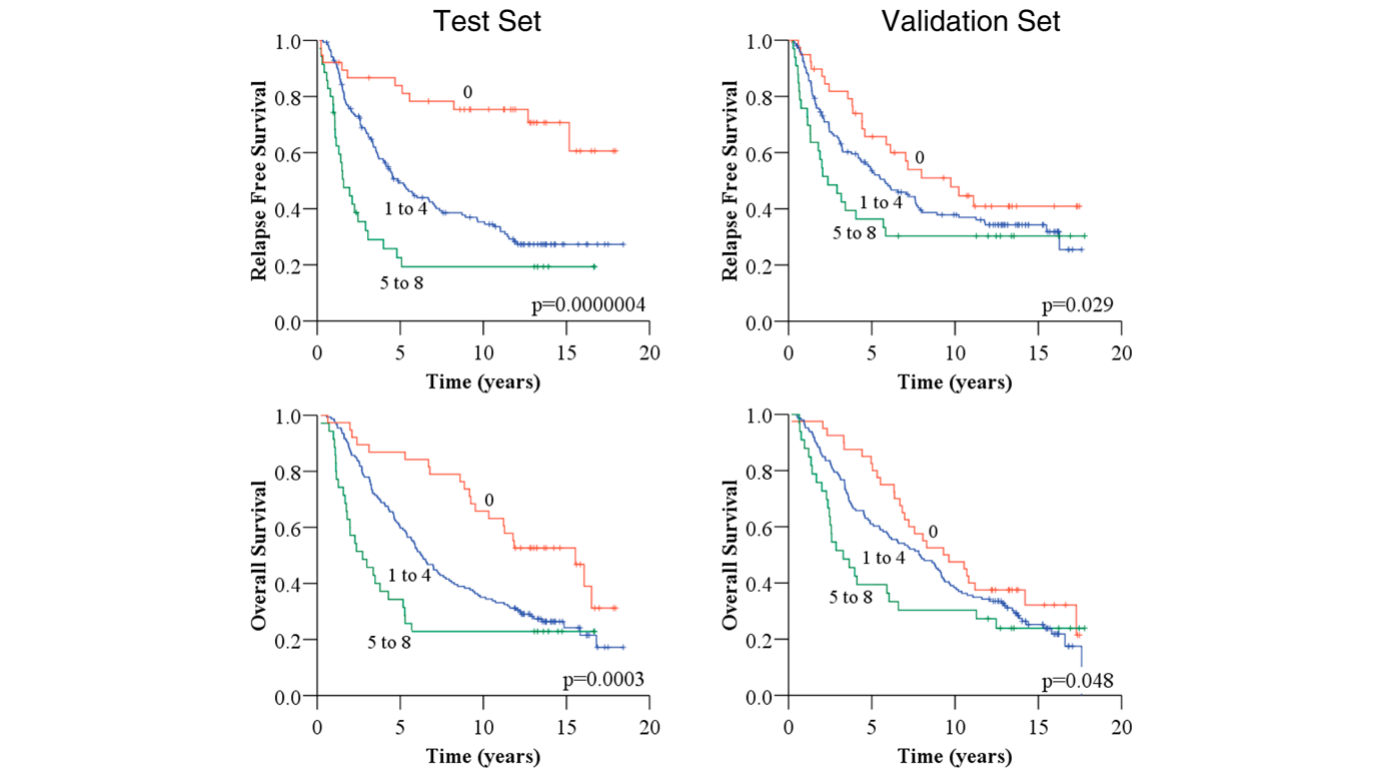
Relapse-free and overall survival by banded biomarker score in the test and validation sets. For each patient, scores for eight immunohistochemical biomarkers assessed were determined; each biomarker was scored as 1 if predicting poor prognosis in univariate analysis for that patient. Patients were then banded by scores of 0, 1 to 4, and 5 to 8. *P *values were obtained by log rank test.

To investigate the ability to stratify patients into prognostic groups by using these biomarkers, a scoring system based on immunohistochemical scores was created to define prognostic subgroups within the test set. Among the 227 patients with scores for all eight biomarkers in the test set, we scored the dichotomized outcome for each marker as 0 for good prognosis and 1 for poor prognosis with respect to univariate analysis of RFS outcomes (that is, 1 each if ER negative or PR negative, and 1 each if positive with respect to the other six biomarkers). Each patient was therefore assigned a score from 0 to 8. Patients were then banded by this score into three groups based on scores of 0, 1 to 4, or 5 to 8. Banding was performed without assumption regarding the relative importance of each marker or weighting to any one in particular and was defined prospectively. In considering the use of adjuvant chemotherapy for these three scoring groups, an imbalance was seen with use in 35.1%, 52.6% and 73.0% of the 0, 1 to 4, and 5 to 8 scoring groups, respectively. RFS outcomes for the three banded groups were markedly different within the test set (Figure 1 and Table 6). The subgroup scoring 0 for all eight biomarkers (38 patients, 16.7%) had 10-year RFS of 75.4% (SEM 7.1%) with a median not yet reached at a median follow-up of 11.7 years. By comparison, the groups scoring 1 to 4 (154 patients, 67.8%) and 5 to 8 (35 patients, 15.4%) had 10-year RFS rates of 35.3% (SEM 4.1%) and 19.3% (SEM 7.0%), and median RFS of 4.8 years (95% CI 3.6 to 6.1) and 1.6 years (95% CI 0.8 to 2.3), respectively. Similar differences in median and 10-year outcomes were also seen with respect to overall survival (Figure 1 and Table 6), which again determined good outcome for the group scoring 0 for all eight markers.

**Table 6:** Relapse-free and overall survival with respect to biomarker score for test and validation sets

Survival	Division	Test set			Validation set		
		*n*	Median RFS, years (95% CI)	10-year RFS, percentage (SEM)	*n*	Median RFS, years (95% CI)	10 year RFS, percentage (SEM)
RFS	Overall cohort	313	5.2 (3.9–6.5)	38.3 (2.9)	289	5.8 (4.4–7.3)	38.1 (2.9)
	0	38/227	NR	75.4 (7.1)	40/219	9.7 (5.5–14.0)	47.8 (8.4)
	1–4	154/227	4.8 (3.6–6.1)	35.3 (4.1)	146/219	5.8 (3.9–7.7)	37.8 (4.2)
	5–8	35/227	1.6 (0.8–2.3)	19.3 (7.0)	33/219	2.4 (0.9–3.8)	30.3 (8.0)
	All markers	227/227	5.2 (3.6–6.9)	39.8 (3.4)	219/219	5.8 (4.2–7.5)	38.5 (3.4)
OS	Overall cohort	313	6.7 (5.7–7.8)	39.5 (3.4)	289	7.3 (5.9–8.6)	39.1 (2.9)
	0	38/227	15.5 (10.9–20.1)	67.6 (7.7)	40/219	9.3 (5.5–13.1)	47.5 (7.9)
	1–4	154/227	6.4 (5.3–7.5)	35.1 (3.8)	146/219	7.8 (5.8–9.9)	38.4 (4.0)
	5–8	35/227	2.7 (1.1–4.3)	21.6 (6.8)	33/219	3.3 (1.7–4.9)	30.3 (8.0)
	All markers	227/227	6.6 (5.2–8.0)	38.2 (3.2)	219/219	7.6 (6.0–9.1)	38.8 (3.3)

Relapse-free and overall survival are presented in the test and validation sets for the overall cohorts, for the subsets with expression scores available for all eight biomarkers assessed and with respect to patient subgroups for biomarker scores of 0, 1 to 4, and 5 to 8. RFS, relapse-free survival; OS, overall survival; NR, not reached; CI, confidence interval.

The same analysis was repeated in the validation set with respect to this scoring system. OS and RFS by Kaplan–Meier analysis demonstrated statistically significant differences between the prognostic subgroups; however, the difference in survival outcomes was less marked between the prognostic groups compared with the test set (Figure 1). Confidence intervals overlapped for both RFS and OS for the groups scoring 0 and 1 to 4 but were non-overlapping between the groups scoring 1 to 4 and 5 to 8 (Table 6).

**Figure 2:**
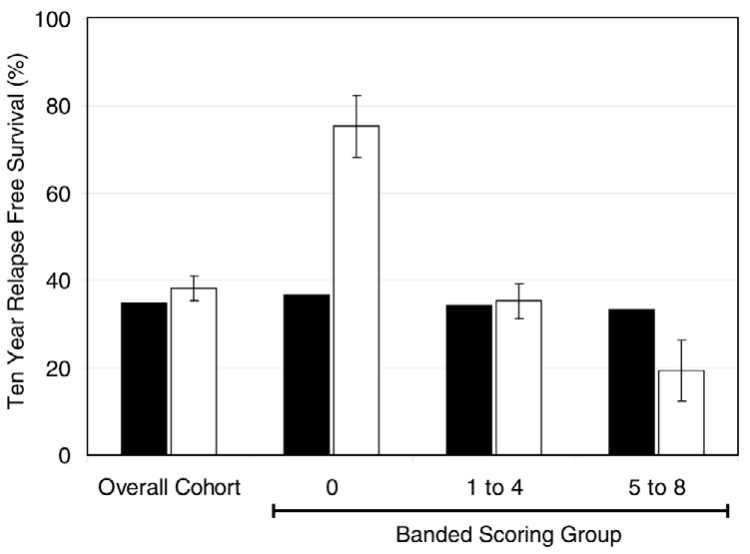
Comparison of mean predictions for relapse-free survival by Adjuvant! with actual outcomes. Predicted outcome for percentage relapse-free survival at 10 years for each patient, based on their baseline clinical and pathological factors, was determined with the online prognostic tool Adjuvant!. The means of these predicted outcomes (black bars) are shown compared with the actual outcomes determined by Kaplan–Meier analysis (white bars, ± SEM) for the complete 313-patient cohort in the test set and with respect to patients subgrouped by banded biomarker score for the eight immunohistochemical biomarkers assessed in this study.

After this, we compared actual RFS outcome within the test set of each banded group with the mean of the predictions for 10-year RFS outcomes determined by the online prognostic tool Adjuvant! [27,28]. This program uses conventional prognostic factors of age, comorbidity, ER status, grade, tumor size and number of positive nodes and provides an estimated outcome with respect to different options for adjuvant systemic therapies. Consistent with previous validation of Adjuvant! in a large population-based cohort [26], mean predicted values for RFS at 10 years agreed closely with actual outcomes determined by Kaplan–Meier analysis in the overall 313-patient cohort (Figure 2) and additionally for the 227-patient subgroup with scores for all eight biomarkers (data not shown). In contrast, for the good-prognosis subgroup scoring zero for all eight biomarkers, the mean of predictions for percentage 10-year RFS by Adjuvant! was 36.7%, but a better actual outcome of 75.4% (SEM 7.1%) was in fact observed. Values for the 5 to 8 biomarker score group were 33.4% and 19.3% (SEM 7.0%), respectively, indicating an actual outcome that was worse in this group than predicted by Adjuvant!. By comparison, values for the intermediate group scoring 1 to 4 were similar at 34.4% and 35.3% (SEM 4.1), respectively.

Having seen a less impressive distinction between prognostic groups with our biomarker scoring system in the validation set, we performed exploratory multivariate analysis using the combined test and validation sets inclusive of baseline prognostic factors and each TMA biomarker. Similarly to the results in the test set, tumor size, percentage of positive axillary nodes and the TMA biomarkers PR, Ki-67 and CA IX each maintained independent prognostic significance with respect to RFS (Table 7).

**Table 7:** Multivariate analysis of relapse-free survival in the combined 602-patient cohort

Factor	Hazard ratio (95% CI)	*P*
Tumor grade (3 versus 1 or 2)	1.18 (0.88–1.57)	0.3
Tumor size, cm		0.02
>2–5 versus 0–2	1.49 (1.10–2.00)	0.009
>5 versus 0–2	1.63 (1.09–2.46)	0.02
Lymphovascular invasion	1.13 (0.78–1.65)	0.5
Percentage of positive nodes		0.001
>50–99 versus 0–50	1.25 (0.94–1.66)	0.1
100 versus 0–50	1.90 (1.35–2.71)	0.003
ER	0.91 (0.63–1.32)	0.6
PR	0.72 (0.54–0.96)	0.02
HER2	1.22 (0.89–1.66)	0.2
EGFR	1.13 (0.71–1.80)	0.6
Ki-67	1.39 (1.05–1.84)	0.02
p53	1.00 (0.74–1.35)	0.99
CA IX	1.58 (1.12–2.22)	0.008
CK5/6	0.98 (0.61–1.57)	0.9

ER, estrogen receptor; PR, progesterone receptor; HER2, human epidermal growth factor receptor 2; EGFR, human epidermal growth factor receptor 1; CA IX, carbonic anhydrase IX, CK5/6, cytokeratin 5/6; CI, confidence interval.

## Discussion

Early breast cancer involving four or more axillary nodes carries a poor prognosis; however, a proportion of patients do well and are cured of their disease. Ten-year RFS rates of 38% seen in this study mirror those from historical series [4]. Management decisions might be improved if prognostic subgroups can be identified.

We first investigated current conventional prognostic factors for breast cancer to assess their ability to determine prognosis in such patients. Tumor size and percentage of positive axillary nodes were the most important factors, with each retaining prognostic significance in multivariate analysis, consistent with previous data for each [4,29]. Both reflect overall tumor burden at diagnosis, increasing risk of occult metastatic disease at diagnosis and issues of surgical resectability. Additionally, we addressed the utility of eight biomarkers to determine prognosis in this group. Of these, PR, Ki-67 and CA IX retained prognostic significance after multivariate analysis that included conventional prognostic factors. The biological relevance of each in determining prognosis must remain somewhat speculative. PR might be important in prognostication in luminal-type subclasses, which remain an indistinct area of breast cancer molecular subtype classification. PR expression may show independent prognostic value (in addition to molecular markers for genomic grade) in ER-positive breast cancers, and this seems to be mirrored in our study for heavily node-positive disease [13]. Ki-67 is probably represented here as a marker of tumor proliferation relating to intrinsic phenotypic aggressiveness, risk of occult metastatic disease and as a predictive factor for responsiveness to systemic therapies. Finally, the hypoxia-inducible gene CA IX is an established and validated poor prognostic factor in breast cancer [23,30]. Its precise function remains inadequately determined and so the underlying biological explanation for its independent prognostic value in this cohort remains to be fully explained.

Our attempt to develop a prospectively defined prognostic scoring system, based on immunohistochemical biomarkers, for this patient group resulted in marked separation in survival outcomes within the test set cohort. In the validation set, cohort distinction in outcomes with this scoring system, although retaining statistical significance, indicated smaller differences between subgroups. This approach would therefore seem to be an imperfect method of predicting differential outcomes among those with four or more positive axillary nodes, and the scoring method described here requires refinement. Our results do, however, indicate that conventional baseline prognostic factors can be usefully augmented by the addition of information derived from molecular biomarkers in patients with heavy axillary nodal involvement, who as a group have received less attention in the age of molecular breast cancer subtyping. Options for refinement of our approach might include incorporation of other biomarkers that have been shown to predict prognosis independently of conventional prognostic factors for breast cancer, for example Bcl-2 [31]. Alternatives to immunohistochemistry for detection and expression analysis of relevant prognostic genes may also be appropriate; for example, analysis by array-comparative genomic hybridization, cDNA microarray and RT-PCR approaches have each been shown to permit prognostic classification [11,12,16-19,21,32-34]. The most appropriate methodology for subsequent application in the clinic has yet to be defined.

The finding in the test set that the Adjuvant! online prognostic tool predicted accurately for the overall group but did not discriminate those within different prognostic groups argues for the validation of molecular markers that can enhance such a mathematical model to individualize prognostic information further and be more sensitive to the heterogeneity of the disease. Such approaches are being prospectively tested in the axillary node negative setting [1,17-21] and we believe they also hold promise in those with heavy axillary nodal involvement.

Our internal validation approach represents one option for exploratory testing and subsequent confirmation of experimental prognostic methodologies. It is widely accepted that validation in independent cohorts, from those in which a model is originally derived, is a mandatory step in the development of prognostic methods. However, no clear consensus exists on the most robust internal method of undertaking this. Others have advocated alternatives to our straightforward approach of randomization to two cohorts, such as dividing data in a non-random way (for example by time period of patient presentation) or the use of bootstrapping or 'leave one out' cross-validation approaches [35]. The gold standard remains external validation by separate investigators, but this leaves the issue of how best to first internally validate findings.

With respect to potential limitations of our study, our TMA cohort includes patients presenting between 1986 and 1992, who were treated in accordance with therapeutic strategies that have since evolved. Overall, the figure of only 50.5% receiving chemotherapy in the test set is significantly lower than would be expected for this patient group in the modern era. Furthermore, no patients received certain treatments options that are now standard, such as trastuzumab or taxanes. However, we believe our conclusions remain valid, for three reasons. First, we have found a difference in the use of chemotherapy between the three prognostic groups created by the novel scoring system developed in our study (35.1%, 52.6% and 73.0% of the 0, 1 to 4 and 5 to 8 scoring groups, respectively). If one assumes that the chemotherapy will have improved outcome in the three respective groups, then the imbalance would in fact have biased against seeing a difference in the three prognostic groups we had created. The use of RFS as an outcome might be affected by treatment imbalance. However, we have also provided data for overall survival that essentially showed similar, if less marked, findings for outcomes with respect to the three prognostic divisions created. Second, since the mid 1970s, the British Columbia Cancer Agency has periodically circulated updated consensus provincial practice guidelines to all physicians in the province. Published data from the time span of this study confirm that the degree of compliance with provincial practice guideline recommendations for radiotherapy, chemotherapy and tamoxifen was high [36]. We believe that the same excellence for achieving management standards in the heavily node-positive disease cohort considered here can be assumed. Third, this large cohort, derived from a TMA including more than 4,400 patients and from within a single healthcare setting, comprises patients presenting during the period 1986 to 1992 and is 'population based' by nature, which is a strength of the data set. Chemotherapy should therefore be expected to be a less commonly used modality. A further question with regard to TMA-based biomarker studies is the quality of the pathological samples available and the concordance between the eligible patient cohort and those with scorable results for marker(s) of interest. In our study all eight biomarkers were scored in 227 of 313 patients in the test set cohort. Baseline pathological characteristics and survival outcomes were not significantly different in this subgroup from those in the overall group. Thus, our biomarker data are likely to be representative of the group as a whole.

## Conclusion

This study demonstrates that conventional prognostic factors of tumor size and the percentage of positive axillary nodes, together with biomarkers of PR, Ki-67 and CA IX, are independent prognostic factors in breast cancer patients with four or more positive axillary lymph nodes. Our prognostic scoring system, based on the expression of eight biomarkers, identified markedly different survival outcomes in the test set, with less marked but statistically significant differences in the validation set. This study highlights the importance of validation of initial findings. Further investigation is warranted to determine how prognostic stratification can best be evolved to incorporate biomarkers to permit the development of more tailored therapeutic decision making for this patient group.

## Abbreviations

CA IX = carbonic anhydrase IX; CI = confidence interval; CK5/6 = cytokeratin 5/6; EGFR = human epidermal growth factor receptor 1; ER = estrogen receptor; FISH = fluorescence *in situ *hybridization; HER2 = human epidermal growth factor receptor 2; OS = overall survival; PR = progesterone receptor; RFS = relapse-free survival; RT-PCR = polymerase chain reaction with reverse transcription; TMA = tissue microarray.

## Competing interests

The authors declare that they have no competing interests.

## Authors' contributions

SC conceived the design of the biomarker scoring system, performed the statistical analysis in the test set, the data collection and analysis from Adjuvant!, and drafted the first version of the manuscript. CDB assisted with the design of the scoring system, oversaw the statistical analysis applied to the test and validation sets and revised the statistical content of the manuscript. SL performed the statistical analysis in the validation set. CHS collected and performed analysis of the patient clinical and outcomes data linked to the tissue microarray. HK was involved in the design, analysis and interpretation of the data and in significant revision of the intellectual content of the manuscript. DH oversaw the construction, immunohistochemistry and scoring of the tissue microarray and was involved in the design, analysis and interpretation of the data and in revising the intellectual content of the manuscript. KAG conceived the overall design of the study and the analysis and interpretation of the data, and revised the intellectual content of the manuscript. All authors read and approved the final manuscript.
